# When the Past Is Backward and the Future Is Forward: An Embodied Cognition Intervention in Preschoolers with Developmental Language Disorder

**DOI:** 10.3390/jintelligence13060061

**Published:** 2025-05-25

**Authors:** Carla Vergara, Mabel Urrutia, Alberto Domínguez

**Affiliations:** 1 Facultad de Ciencias Sociales, Universidad de Concepción, Concepcion 4070386, Chile; carlamvergara@udec.cl; 2 Facultad de Comunicaciones y Artes, Universidad de Las Américas, Concepcion 4030000, Chile; 3 Facultad de Educación, Universidad de Concepción, Concepcion 4070386, Chile; 4 Instituto Universitario de Neurociencia (IUNE), Universidad de La Laguna, Campus Guajara, 38205 La Laguna, Tenerife, Spain; adomin@ull.es

**Keywords:** developmental language disorder, embodied cognition, conceptual metaphor, motor training, induced plasticity technique

## Abstract

This quasi-experimental study investigates the impact of an embodied intervention on the semantics of transitive verbs in children with Developmental Language Disorder (DLD), grounded in the “TIME IS SPACE” conceptual metaphor—where the future is mapped as forward and the past as backward. The intervention involved a pretest and a posttest design, using the induced plasticity technique to saturate motor areas through repetitive arm movements (either forward or backward). Then, we determined the influence of this saturation on the auditory comprehension of past- and future-tense sentences. Fifty-seven children (ages 5 years and 6 months to 6 years and 9 months) participated in the experiment. Participants were divided into four groups: two groups of children with DLD—14 Chilean students from speech therapy institutions who received the intervention and 15 who did not—and two groups of chronologically matched typically developing (TD) peers, with 14 children in each intervention condition. The hypothesis proposed that a psychoeducational intervention would enhance the comprehension of time–space conceptual metaphors in children with DLD, reflected by greater interference effects (higher RTs and lower ARs in matching vs. mismatching conditions). A 2 × 2 × 2 × 2 × 2 mixed ANOVA was used to identify significant differences in reaction times and accuracy rates. Results showed significant differences in the posttest for the DLD group with intervention versus the same group without intervention, particularly in the semantics of future tense with forward motion. Furthermore, the study found that the impact of the intervention depended on the level of narrative discourse comprehension. These findings suggest that embodied interventions leveraging metaphorical mappings of time and space can enhance verb tense comprehension, particularly in preschoolers with narrative comprehension challenges.

## 1. Introduction

Children’s language development involves not only the acquisition of grammatical and lexical structures but also the ability to comprehend figurative meanings, such as those conveyed through metaphorical expressions. This poses a particular challenge for children with Developmental Language Disorder (DLD), a condition that affects multiple levels of language and is commonly associated with semantic, discursive, and motor difficulties (Ahufinger et al. 2021; Bishop et al. 2017; Sanjeevan and Mainela-Arnold 2019). These deficits hinder the ability to infer figurative meanings (Andreou et al. 2022; Bühler et al. 2018) and to maintain narrative coherence in discourse comprehension (Delgado-Cruz et al. 2022; Winters et al. 2022).

In this context, embodied metaphors offer a novel approach by linking linguistic comprehension to sensorimotor experiences. From a developmental standpoint, such difficulties should be examined in relation to key developmental milestones (Conti-Ramsden and Durkin 2017) since the ability to interpret and comprehend language emerges gradually as semantic and cognitive abilities such as categorization and abstraction consolidate (Dauvister et al. 2022; Ruiz et al. 2021). These skills are frequently weaker in children with DLD.

Child language development is closely linked to motor progress, particularly fine motor control and interaction with the environment, which facilitate the acquisition of vocabulary and early communicative skills (Lockman and Tamis-LeMonda 2020). Between the ages of three and four, children begin to produce more complex utterances, characterized by the use of morphological inflections, such as verb tense (Hadley 2020). From ages four to six, children develop an understanding of figurative language and abstract vocabulary (Zhu et al. 2024), while morphosyntactic structures—including the use of subordinate clauses and verb conjugations—continue to increase in complexity (Leonard 2014). In children with Developmental Language Disorder (DLD), these developmental milestones often appear later or follow atypical trajectories, underscoring the need for assessments and interventions tailored to the developmental profile of this population (Bishop et al. 2017; Dauvister et al. 2022).

In comprehension, semantic deficits in DLD are reflected in difficulties when inferring contextual meaning (Dawes et al. 2019; McGregor et al. 2024) and when maintaining cohesion in discourse comprehension (Andreu et al. 2021; Lin et al. 2024). In expression, children with DLD tend to have a limited vocabulary and build less complex sentences compared to their typically developing (TD) peers (Leonard et al. 2019; Finestack 2018). Comprehension difficulties are associated with executive dysfunctions affecting both verbal and non-verbal tasks (Blom and Boerma 2020; Kapa and Erikson 2020; Larson et al. 2020), as opposed to expressive difficulties, which are limited to verbal tasks (Acosta et al. 2017).

Among the non-linguistic difficulties associated with the disorder, Reggin et al. (2023) note a high comorbidity between DLD and developmental coordination disorder (DCD). The longitudinal study by Sack et al. (2021) revealed that certain early fine and gross motor deficits among preschool children with DLD can predict persistent language impairment in the future. These studies highlight the need to analyze whether an embodied and situated context might benefit the DLD population, as motor deficits are likely related to the language difficulties associated with the disorder.

The effectiveness of interventions on children with DLD and semantic comprehension difficulties has not yet been sufficiently addressed through empirical research. Likewise, available results are not conclusive so far given the heterogeneity of symptoms associated with the disorder (Acosta et al. 2017; American Psychiatric Association 2013; Rinaldi et al. 2021). For instance, Dam et al. (2020) evaluated an intervention that used related words in order to improve the vocabulary of bilingual children with TD and DLD. While they found no significant changes in the naming task in English, they did find a positive correlation between vocabulary knowledge in Spanish and English in the posttest. These results suggest that, even though the strategies can improve vocabulary, they might be insufficient to help the children completely overcome the difficulties of DLD (Finestack 2018).

Different studies highlight the importance of developing interventions individualized that address the complex linguistic and non-linguistic needs of children with DLD (Dawes et al. 2019; Delgado-Cruz et al. 2022; Ramírez et al. 2023). Simulating perceptual and motor experiences is an effective way to support the learning of complex processes and enhance comprehension (Johnson-Glenberg and Megowan-Romanowicz 2017; Johnson-Glenberg et al. 2016; Loeffler et al. 2020). These studies are grounded in embodied cognition theory, which posits that cognitive processes, including language, are closely linked to perception and action, since understanding arises from the activation of prior sensorimotor experiences (de Vega 2021; Zwaan and Taylor 2006).

Glenberg and Kaschak (2002) demonstrated this connection through the Action–Sentence Compatibility Effect (ACE), showing that sentence comprehension is influenced by the congruence between the meaning of the sentence and the bodily movement. This idea aligns with the simulation model proposed by Barsalou (2008), which posits that those concepts—even abstract ones—are represented through situated simulations grounded in perceptual and motor experiences. In the field of embodied metaphors, the Conceptual Metaphor Theory developed by Lakoff and Johnson (1980, 1999) argues that abstract structures are understood through concrete experiences, as exemplified by the TIME IS SPACE metaphor. Núñez and Cooperrider (2013) expand on this notion by distinguishing two variants of the metaphor, Ego Moving and Time Moving, both reflecting spatial mental models of time. Casasanto has further shown that such metaphors have functional implications for learning; for example, physically placing positive stimuli in an upper position—in line with the “good is up” metaphor—enhances word meaning retention (Casasanto and de Bruin 2019).

There is also evidence supporting the integration of perceptual and motor experiences into the learning process to enhance comprehension and knowledge retention. In this regard, de Koning et al. (2017) examined the effects of a sensorimotor simulation-based training on reading comprehension in third- and fourth-grade children, concluding that incorporating such experiences during reading significantly improves comprehension in younger learners. Similarly, the study by Johnson-Glenberg et al. (2016), focused on embodied cognition and the learning of centripetal force concepts, showed that the level of embodiment during learning sessions interacted significantly with time. Specifically, participants exposed to high-embodiment conditions performed better on generative knowledge questions, suggesting that greater bodily engagement during the encoding phase supports the retention of complex types of knowledge. However, despite growing evidence supporting embodied cognition, studies evaluating interventions based on this approach remain limited—particularly among children, and even more so among those with learning difficulties.

In light of the existing gaps in DLD research and the findings on embodied cognition in language learning (Demir and Goldin-Meadow 2016; Kosmas and Zaphiris 2020), the present study adopts an embodied cognition approach within the framework of the semantic resonance model (Bidet-Ildei et al. 2020) to examine semantic impairments in individuals with DLD. This model posits that the activation of common semantic representations does not necessarily depend on the activation of specific motor components. According to Bidet-Ildei et al. (2020), while motor components may contribute to the semantic processing of verbs, their involvement depends on whether the action is inherently embedded in the word’s meaning (Kemmerer et al. 2008; Yang et al. 2017). Specifically, when the meaning of a word entails an action, motor components play a crucial role in establishing the functional link between action representation and the processing of action-related words (Pulvermüller 2005; Zwaan and Taylor 2006).

Our study used Ruiz et al.’s (2021) experiment on locomotive comprehension of time as its foundation. Ruiz et al.’s work seems to be the only research that uses mechanisms based on embodied cognition to analyze semantic comprehension in children with DLD. The authors used the conceptual metaphor ‘TIME IS SPACE’ for the comprehension of tense through the use of space (Casasanto and de Bruin 2019; Gijssels and Casasanto 2017; Spatola et al. 2018). This conceptualizes the past as ‘backward’ and the future as ‘forward’ within an embodied approach by moving the right hand forward and backward. In this context, hand movement transfer refers to the act of moving the hand in specific directions to represent time, using physical space to activate temporal representations (for examples of the metaphor, see Casasanto and Boroditsky 2008; Beracci and Fabbri 2024). This type of movement facilitates the association between motor experiences and the abstract understanding of time, thereby promoting a greater integration of temporal concepts at both cognitive and sensorimotor levels.

In this regard, the work of Gijssels and Casasanto (2017) on spatiotemporal metaphors and their correspondence with sensorimotor responses demonstrated that individuals activate antero-posterior sequences along the sagittal axis during the linguistic processing of past and future verbs. Specifically, participants showed a tendency to associate past events with retraction (backward) movements and future events with advancement (forward) movements, both in motor response tasks and semantic judgments. Responses were faster when the movement direction was consistent with the verb semantics, as observed in experimental conditions such as past–backward and future–forward.

Previous studies (Berchicci et al. 2020; Marrero et al. 2015, 2023) have shown that preparation for bodily locomotion involves high cognitive processing, while the direction of movement reveals distinct cortical organization and functional specialization. In an EEG study, Berchicci et al. (2020) demonstrated that moving backward requires higher cognitive control and can be considered avoidance behavior, while moving forward aligns with an action-oriented behavior, which is primarily located in parietal areas. Similarly, Marrero et al. (2023) found that words associated with approach attitudes (positive valence) may facilitate forward movements, whereas words related to avoidance attitudes (negative valence) may facilitate backward movements.

In the study by Ruiz et al. (2021), hand movement transfer used the induced plasticity experimental technique (Casasanto and Chrysikou 2011; Glenberg et al. 2008). This technique involves causing motor saturation of the transfer effector related to the semantic aspect in verb comprehension by repeatedly presenting a stimulus or task. In this context, the ‘TIME IS SPACE’ metaphor serves as a valuable tool for understanding the complex process of semantic verb comprehension in this population. From a pragmatic perspective, metaphors have a dual meaning. On the one hand, they convey a literal sense related to the physical dimension of spatial displacement; on the other, their figurative interpretation relates to the cognitive structuring of time in terms of movement. This dual nature requires skills such as inferring implicit meanings, establishing conceptual connections, and adapting linguistic interpretation to the context (Casasanto and de Bruin 2019). In this regard, difficulties in metaphor comprehension can adversely affect the acquisition of reading skills as they limit the interpretation of texts containing figurative expressions and hinder the development of global discourse comprehension abilities (de Koning et al. 2017).

This approach enables the exploration of a relatively understudied area in the literature, particularly considering that the effectiveness of interventions for children with DLD remains inconclusive regarding the semantic subtype (Acosta et al. 2017; Rinaldi et al. 2021). This lack of consensus may be partly due to the heterogeneity of the disorder’s clinical set of symptoms (American Psychiatric Association 2013), which makes the application of standardized approaches more complex and contributes to variability in intervention outcomes.

The research by Ruiz et al. (2021) represents a significant contribution to the fields of embodied cognition, metaphor, and DLD due to its novelty and the exploration of a scarcely studied triad. However, its approach was only exploratory. The authors reported facilitation effects on verbal comprehension in participants with DLD, which differed from those observed in typically developing (TD) individuals. These results served as a baseline for developing strategies to enhance both semantic skills and the understanding of verb tenses in individuals with this disorder.

Based on those results, the present study seeks to replicate and expand the experimental approach of Ruiz et al. (2021) by incorporating a psychoeducational intervention. Furthermore, it includes pretest and posttest experimental sessions to assess the effects of the intervention on the comprehension of verbs in the past and future tenses. Thus, this study aims to provide a more comprehensive perspective on education through the design and implementation of an intervention, addressing both the theoretical and practical aspects required to advance the understanding and treatment of semantic deficits in DLD.

In light of the above, we designed an intervention program with the aim of addressing comprehension difficulties of past and future tenses in children with DLD. This intervention included strategies based on semantic resonance from embodied cognition and the ‘TIME IS SPACE’ metaphor. In this context, we replicated the induced plasticity experiment by Ruiz et al. (2021) in order to analyze its effects on TD and DLD children, both before and after the psychoeducational intervention.

The aim of this study was to analyze the impact of an intervention program based on embodied cognition and the metaphorical semantics of transitive verbs on children with DLD, through the use of an induced plasticity technique. We hypothesized that children with DLD would show improvements in the comprehension of conceptual metaphors related to time and space, by means of an induced plasticity experiment after a psychoeducational intervention. This will be evidenced by interference effects in matching metaphor conditions (past–backward, future–forward) and facilitation effects in mismatching metaphor conditions (past–forward, future–backward) through the implementation of an induced plasticity experiment following a psychoeducational intervention.

In studies using other ACE experimental designs, action sentences prime real movement while simultaneously influencing comprehension (Wang et al. 2019; Winter et al. 2022). In these cases, facilitation effects are expected when the movement aligns with the direction described in the sentence (matching conditions), whereas interference effects occur when the movement contradicts the described direction (mismatching conditions). However, when applying the induced plasticity technique, the expected outcomes are reversed, as motor effector saturation is induced through the continuous execution of a movement before linguistic processing (Casasanto and Chrysikou 2011; Glenberg et al. 2008).

Thus, in matching conditions, where the verb semantics and the hand movement are compatible, interference effects are expected due to competition for attentional resources (Glenberg et al. 2008). Specifically, the comprehension of past and future verbs engages neural circuits similar to those involved in action processing for backward and forward movements. Conversely, in mismatching conditions, where the verb semantics and the hand movement are incompatible, facilitation effects are expected as there is no competition between the attentional resources required for linguistic comprehension and those involved in sensorimotor processing.

## 2. Methods

**Figure 1:**
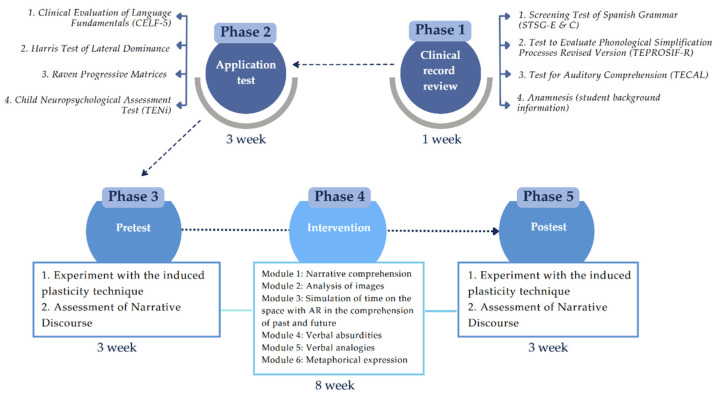
Summary of the study’s methodology. Note: The summary shows the phases of the study. Phases 1 and 2 are initial phases, before the implementation of the experiment and the intervention. The experiment with the induced plasticity technique took place in phases 3 and 5, including the pretest and posttests, respectively. The questionnaire assessing narrative discourse comprehension (EDNA test) was also applied in phases 3 and 5 in order to analyze the effect of the mobile application on this covariate. This took 15 min per participant. The psychoeducational intervention (phase 4) took place in 6 modules. Source: prepared by the authors.

The study had a quasi-experimental, mixed factorial design 2 × 2 × 2 × 2 × 2. The sample consisted of Chilean preschoolers with DLD or typical development. The methodology included standardized assessments, a two-phase experimental task (pretest and posttest), and a psychoeducational intervention delivered through a mobile application. The procedure included diagnosis confirmation, informed consent, and a three-phase design (pretest, intervention, posttest). Data were analyzed through cleaning, normalization, and outlier removal. Methodological details are presented in Figure 1.

### 2.1. Design

The design included 2 between-group independent variables: diagnosis (presence or absence of DLD) and embodied intervention (with or without intervention); and 3 within-group independent variables: verb semantics (past tense, future tense), right-hand motion transfer (forward, backward) and session (pretest or posttest).

### 2.2. Participants

The participants were Chilean students attending speech schools (or speech therapy institutions). Children were paired by gender and had similar social–economic backgrounds, as the totality of the sample lived in urban areas with a high vulnerability index.

Inclusion criteria for the DLD group were ages between 5 and 6 years, right lateral dominance, and a DLD diagnosis as per the scores in accordance with Chilean Decree Number 170/2010 and a classification of semantics-specific difficulties for DLD according to the CELF-5 test. The inclusion criteria for the control group were ages between 5 and 6 years, right lateral dominance, and a score that evidenced DLD in accordance with Chilean Decree Number 170/2010 and CELF-5 scores.

As exclusion criteria, the study considered a non-verbal IQ lower than 70 (see Bishop et al.’s 2017 criteria), first language other than Chilean Spanish, sensory–motor difficulties, intellectual disabilities and/or neurocognitive disorders such as dyslexia and aphasia, among others.

Sample size was calculated by means of an a priori analysis with G*power 3.1.1 software (Faul et al. 2009). Statistical analysis showed a minimum sample size of 12 participants per group with a significance level of α = 0.05, effect size η^2^ = 0.04 and power 1 − β = 0.90. As suggested by García-García et al. (2013), the sample size was adjusted so that the results were not affected by a potential reduction or drop-out of participants. As a result, the sample size was determined to be 15 participants per group, considering a 20% drop-out rate.

The study started with 62 total participants. However, 5 children dropped out of the experiment, meaning the final number of participants was 57 (see Table 1 for descriptive statistics). Out of the 5 children that dropped out of the study, 2 changed educational institutions and 3 stopped attending school for extended periods of time or did not return to the educational community.

### 2.3. Materials

Participants were administered the following three standardized tests: the Clini-cal Evaluation of Language Fundamentals—Fifth Edition (CELF-5; Wiig et al. 2013), the Harris Test of Lateral Dominance (Harris 1957), and the Raven Progressive Matrices (Raven 2003). 

The CELF-5 was specifically used to assess semantic comprehension and to sup-port the diagnosis of Developmental Language Disorder (DLD). For this purpose, scores from the Receptive Language Index (RLI) and the Linguistic Context Index (LCI) were analyzed. During their administration, the tests were adapted at the se-mantic (lexical) level in order to reflect Chilean Spanish. The test’s Cronbach’s alpha ranged from α = 0.69 to α = 0.91 in the sub-tests, whereasits composite scores ranged from α = 0.87 to α = 0.95. The Raven Progressive Matrices test was used to assess non-verbal IQ (Cronbach’s alpha α = 0.80). 

Table 2 shows the analysis of the control variables related to the semantic com-prehension level of language (Receptive Language Index—RLI, Linguistic Context In-dex—LCI) and non-verbal IQ.

The Harris Test of Lateral Dominance (Harris 1957) was used to determine lateral dominance. During the administration, participants were asked to simulate actions described in test items. For example, instead of asking “Which hand do you use to hold a pencil?”, children were asked to perform the action (e.g., “Show me how you draw with this pencil”). The observed responses were then compared with participants’ anamnesis, as documented in their preschool clinical records. This procedure allowed for the confirmation or exclusion of right-hand manual dominance in each participant.

In addition to these tests, the Assessment of Narrative Discourse (EDNA) (Pavez et al. 2008) and the TENI (Tenorio-Delgado et al. 2014) tests were administered in order to analyze covariate effects related to narrative discourse comprehension and Executive Functions (EFs), respectively. The EDNA test involves reading three short stories and answering literal and inferential questions based on those stories. The test’s Cronbach’s alpha is α = 0.84. The TENI test is a set of 10 subtests grouped into eight games that were displayed on a touchscreen device. The following subtests were administered: ‘Alternate Universes’ (Cronbach’s alpha α = 0.80) used to evaluate selective attention; ‘Clumsy Mole’ (Cronbach’s alpha α = 0.77) for working memory; and ‘Bzz! INH’ (α = 0.97) to measure inhibition control. Both the EDNA and the TENI tests have been validated for child populations in Chile.

After administering the instruments, participants took the induced plasticity experiment as a pretest. Subsequently, the psychoeducational intervention took place, which lasted 8 weeks in total. Finally, the experiment was applied as a posttest with participants to evaluate the effects of the intervention.

#### 2.3.1. Experiment

The materials, experimental conditions, and procedure were replicated from Ruiz et al. (2021). Following the study, the first experimental stage consisted of the motor training task, which saturates the motor areas. The second stage involved semantic judgment of sentences.

**Figure 2:**
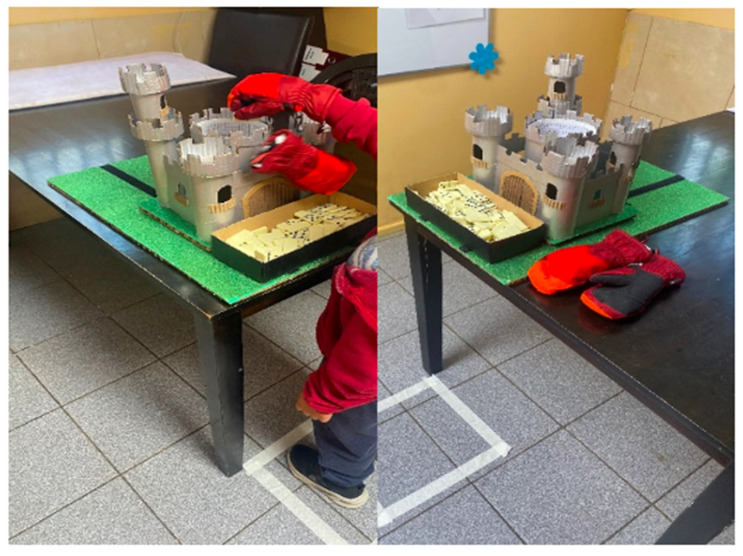
Motor training task and experimental setting.

During the motor training stage, participants listened to a story about a princess who was locked in a haunted castle by an evil wizard. Then, they were asked whether they wanted to help the princess escape. If they agreed to the task and provided assent, they would help to restore the pieces of the castle that were turned into domino tiles. In this fashion, they were requested to return the enchanted tiles by moving a total of 100 pieces with their right hand. The tiles were taken from a box located in the middle of the board and put into the castle tower. Following Ruiz et al. (2021), experiment participants’ arms were measured to control the distance between the box with domino tiles and the castle tower according to the measurements of each child. During the tasks, participants wore a snow glove on their right hand, while the other glove hung from their right wrist. The gloves were used to intensify motor saturation caused by hand motion. The task lasted around 5 min (Figure 2).

Immediately after the motor training task, participants carried out a semantic sentence judgment task. The task consisted of listening to sentences in past and future tense and determining whether they were semantically coherent or not. For this purpose, the E-Prime 3.0 software was used with a QWERTY standard keyboard specifically adapted for the experimental task. Stickers with symbols were added to the ‘6′ and ‘b’ keys (a green ‘correct’ symbol and a red ‘x’, respectively) that participants had to press if the sentence was coherent or not.

**Figure 3:**
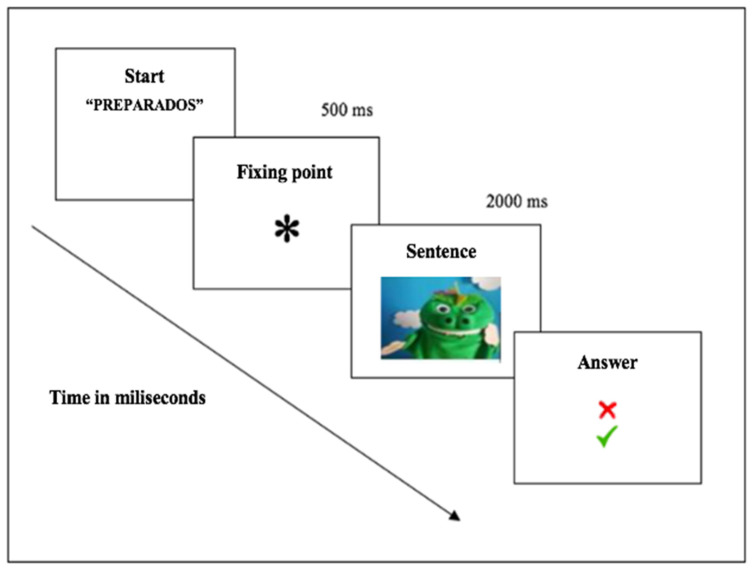
Experimental design from the Ruiz et al. (2021) study.

The instructions for the experiment were presented in a video featuring a dragon that explained what the participants had to do. Children did a practice block of the trials before starting the experiment. After completing the practice block and pressing the space bar on the keyboard, the trials of the experiments started. Each trial began with the presentation of the word “PREPARADOS” (READY, in Spanish) in the center of the screen for 1000 ms, accompanied by an audio that read the word out loud. After this, a fixation asterisk was displayed for 500 ms. Immediately after this, a trial sentence was read out loud by a dragon on screen. This section of the trial lasted approximately 2000 ms. Finally, the ‘correct’ and ‘x’ symbols were displayed until the participant pressed the ‘correct’ or ‘x’ key on the keyboard (Figure 3). After the participant responded, a new trial of the experiment began. The sentence semantic judgment task lasted around 15 min.

While the location of the keys is not a variable in this study, hand movements are. For this reason, the direction of the response was monitored due to its possible in-line effects. In order to do so, the location of the keys was counterbalanced so that the ‘6′ key meant coherent and the ‘b’ key incoherent in half of the trials. Conversely, ‘6′ meant incoherent and ‘b’ was coherent in the remaining half of the trials.

For the semantic judgment tasks, the linguistic material from Ruiz et al. (2021) was used. The stimuli were 54 simple sentences with a transitive verb in second-person singular, a direct object, and a noun or adverbial complement. Out of the 54 sentences, 18 were in future tense (i.e., ‘Tomarás un vaso de leche’; ‘You will drink a glass of milk’ in English), 18 were in past tense (i.e., ‘Tomaste un vaso de leche’; ‘You drank a glass of milk’ in English), and 18 were incoherent (i.e., ‘Tomarás una casa de nubes’, ‘You will drink a house of clouds’ in English). Half of the incoherent sentences were in past tense and the other half in future tense.

#### 2.3.2. Intervention

The psychoeducational intervention was designed especially to address semantic deficits of DLD through strategies based on embodied cognition. To this end, the metaphor ‘TIME IS SPACE’ was employed, as it provides a valuable tool for understanding the complex process of verb comprehension in children with DLD. From a cognitive perspective, the semantics of this metaphor encompass both perceptual and motor components, which are crucial for its interpretation and validate its application in the intervention design. Within this framework, the metaphor’s source domain (SPACE) structures the conceptualization of the target domain (TIME), enabling temporal relationships (the comprehension of abstract concepts) to be understood in terms of spatial displacement (the comprehension of concrete concepts).

During the intervention, participants were instructed to perform specific bodily movements while listening to sentences in different verb tenses. For example, when presented with sentences in the future tense, such as ‘Ayún will give a chocolate to the teacher during recess’, they were asked to perform a forward hand movement. This task reinforced the association between verb semantics and the sensorimotor representation of time, thereby facilitating their comprehension and linguistic processing through the use of metaphor.

The intervention was implemented through a mobile application (app) developed for the Android operating system in the Unity 3d engine and using Vuforia. Additionally, a database was created where reaction times and inaccuracies were recorded to evaluate the progress of participants in each activity. This application stands out from existing solutions as it comprehensively addresses semantic difficulties in verb tense comprehension, using software as a mediating tool for the treatment of this deficit—an approach currently unavailable in the field. Most existing applications primarily focus on phonological aspects (e.g., *Hablando con Nok*) and the pragmatic level of language (e.g., *Conversation Lite*). One application designed for Spanish-speaking children was identified as addressing both semantic and syntactic aspects by incorporating various concepts and semantic categories (professions, transportation, positions, actions, characteristics, and opposites), as well as instruction comprehension and grammatical structures. In addition, this software uses the simulation of motor and perceptual experiences to stimulate language.

**Figure 4:**
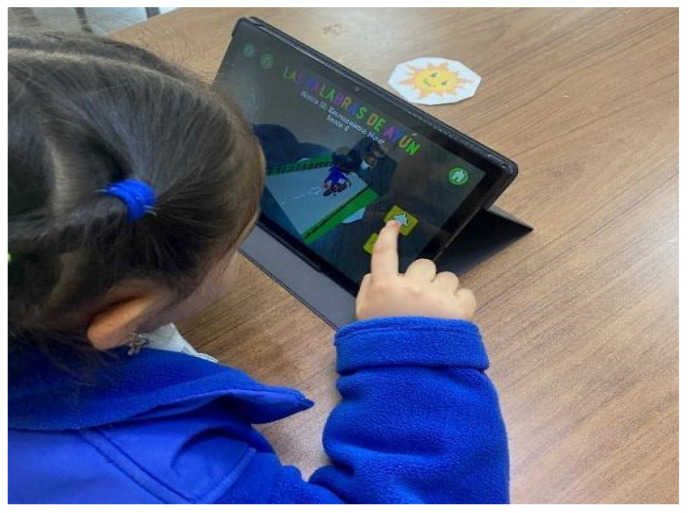
Example of the motor training module using augmented reality (AR).

The app shows the story of Ayún, a 5-year-old virtual character who has difficulties understanding what people say. Ayún is the main character of the app and appears in different sessions, which were created to involve specific aspects of the semantics and pragmatics of verbs. Augmented reality (AR) was utilized to enhance children’s engagement, attention, and motivation, while also serving as a key mechanism to support embodied cognition and address semantic difficulties related to verb tense comprehension. AR allowed children to activate mirror neurons by adopting a second-person perspective (Gallese 2014) and control movement (forward or backward) by performing direct hand movements, which in turn generated the movement transfer central to the experiment. Additionally, the application includes activities related to the semantic interpretation of verbs and reading motivation, as well as image training strategies (Figure 4).

The application follows an intervention model based on a gradual and structured approach, incorporating linguistic, metacognitive, and social components through play (see an example of a similar model in Ramírez et al. 2023). Similar to Acosta et al.’s (2016) ‘Read in a Click’ program, the app-based intervention is organized into levels that adapt to the student’s progress, with increasing complexity in the activities as they advance. The app included a total of 9 sessions grouped in 6 areas. Only Areas 1 and 2 contain more than one session: the first area includes sessions 1 and 2; the second area includes sessions 3, 4, and 5.

The first area of the app focused on narrative comprehension through reading an e-book that included animations and multiple-choice questions which aimed to evaluate literal comprehension and the ability to make inferences. The second area focused on the observation and analysis of images to help users identify and recognize important details. The third area addressed the conceptual metaphor ‘TIME IS SPACE’ through the simulation of spatial displacement with AR, linking the verb tense of sentences with the movement of the main character of the app. The fourth area focused on the analysis of verbal absurdities in order to identify incoherence in language. The fifth area focused on verbal reasoning by means of completing sentences that create analogies. Lastly, the sixth area explored the interpretation of metaphorical expressions by using visual and gestural support in order to enable metaphorical meaning comprehension. The sessions took place within an eight-week period, which allowed for a progressive integration of semantic and pragmatic skills.

The effectiveness of the app was evaluated by analyzing students’ achievement rates in each of the 6 areas of the intervention. Four of the areas demonstrated performance above or equal to 70% among DLD participants. However, ratings in the second and sixth areas were below 60%. In the second area, specific grammatical forms were used in sentence comprehension, such as past tense, which requires a greater ability to understand and apply grammatical rules. Children with DLD may have difficulties with complex verb structures, such as tense changes, making this session more challenging for them. Additionally, the sixth area included sentences with figurative meaning, such as ‘shakes like jelly,’ posing an additional challenge, as children with DLD often struggle to understand double meanings or metaphorical expressions.

Notably, the third area (with AR) included items with high discrimination and low difficulty levels, which represents a particularly advantageous combination, as it facilitates effective differentiation among individuals with varying levels of ability, both above and below the mean. Furthermore, the use of AR was particularly effective in enhancing children’s motivation to complete the task and reducing mental workload (Syaza-Jeffri and Awang-Rambli 2021). By transforming the activity into a game-like experience, the AR environment encouraged participants to simulate the hand movement transfer, thereby engaging perceptual and motor components involved in mapping the embodied metaphor onto the linguistic comprehension of sentences in past and future tense.

### 2.4. Procedures

For this study, several visits to educational institutions took place (two speech schools and a primary school) to review participants’ clinical records, administer evaluation instruments, conduct pretest experiments, implement the psychoeducational intervention, and carry out the posttest experiment. These visits were authorized by the institutions’ directors. Likewise, parents/guardians provided informed consent, and the children provided their assent before each session. All documents were reviewed and approved by The Ethics, Bioethics, and Biosafety Committee of the Vice-Rectory for Research and Development at the Universidad de Concepción (CEBB 1259-2022).

At the beginning of the study (phase 1), the clinical records of the participants were reviewed to confirm that they met the inclusion criteria regarding their diagnosis of DLD. This was confirmed by reviewing the scores of three instruments validated for the Chilean context. Said instruments are administered as per Ministry of Education requirements by speech therapists in educational institutions at the start of the school year. This stage extended for one week. One of these instruments is the Screening Test of Spanish Grammar (STSG-E & C) by A. Toronto, reviewed by Pavez (2010). The test assesses expression and comprehension of grammar. It has a Spearman correlation coefficient ranging from ρ = 0.70 to ρ = 0.83 in its subtests. Also, the Test to Evaluate Phonological Simplification Processes Revised Version (TEPROSIF-R) was used (Pavez et al. 2009), with a Cronbach alpha α = 0.90. Finally, the Test for Auditory Comprehension (TECAL) was used, in its version adapted at Universidad de Chile (Pavez 2008). The reliability of the test was estimated by using the test–retest method, which found no significant differences between the first and second applications of the instrument.

In phase 2, the instruments were applied in a well-ventilated and well-lit room in educational institutions for 3 weeks in total. The experiment took place in phases 3 and 5, the pretest and posttest, respectively. The sessions took approximately 20 min per participant: 5 min for motor training and 15 min for the sentence comprehension task. The questionnaire assessing narrative discourse comprehension (EDNA test) was also applied in phases 3 and 5 in order to analyze the effect of the mobile application on this covariate. This took 15 min per participant. The psychoeducational intervention (phase 4) took place in 9 individual sessions, each taking 25 min per participant. The intervention sessions were conducted twice a week for 8 weeks in total. Both the pretest and posttest phases of the experiment and the individual administration of the EDNA test spanned three weeks, as their implementation depended on the availability of participants and educational institutions.

All phases of the study were conducted through individual sessions led by a PhD student in Psychology. At the start of the interaction with each student, the evaluator requested them to provide their explicit assent by asking if they wanted to do the activity. Children confirmed their interest in participating in the experiment by either nodding or giving a thumbs-up.

### 2.5. Data Analysis

Before the analysis, the data were purged in order to identify and exclude atypical values. First, incoherent sentences were eliminated and excluded from the analysis. Only experimental sentences were included in the analysis. Also, gross reaction times (RTs) were explored in order to observe their distribution and identify atypical data by using histograms and boxplots. The initial preview showed a marked positive asymmetrical distribution. Because of this, log transformations were made in order to normalize the data distribution (Curran-Everett 2018; West 2022). These transformations were made by applying a base 10 logarithm to RTs and accuracy rates (Ars).

While the transformations improved the distribution, the data still had some outliers. To address this issue, Berger and Kiefer’s (2021) and Whelan’s (2008) criteria were used in order to exclude extreme RTs faster than 250 ms and slower than 5000 ms. The purpose of this was to avoid spurious effects related to errors, inconsistent processes, distractions, or severe difficulties in attention that might distort the analysis.

## 3. Results

A repeated-measures ANOVA was used to identify within-subject differences in verb semantics, motion transfer, and session RTs. Additionally, a mixed ANOVA was used to evaluate the main effects on reaction times between groups: both with and without intervention, and between DLD and TD groups. This analysis was replicated for ARs as the dependent variable. The analysis was carried out using SPSS Statistics software version 25.

### 3.1. Confirmation of the Assumptions in ANOVA

A verification was made to find out if the assumptions were confirmed for RTs. In this case, we checked for normality of the data by using the Shapiro–Wilk test. Results showed that the data had a normal distribution in all groups (*p* > 0.05 for each group). The Levene test was not significant (*p* > 0.05 in all comparisons), confirming the homogeneity of variances between groups.

In the case of accuracy, the assumptions were not met, as indicated by the Shapiro–Wilk test (*p* < 0.001). The Levene test was not significant (*p* > 0.05), reflecting homogeneity of variances between groups, which in turn confirms this assumption. Thus, nonparametric tests were applied, as the data did not show a normal distribution.

### 3.2. Nonparametric Tests

Nonparametric Wilcoxon signed-rank tests were conducted for the dependent variable that did not meet the assumptions of normality. An exploratory evaluation of the differences in accuracy without log transformations was carried out for the different evaluation stages (pretest and posttest) and temporal conditions (past–forward, past–backward, future–forward, and future–backward).

In the DLD group without intervention, ARs were significantly higher in the mismatch past condition (i.e., past–forward) when compared to the matching future condition (i.e., future–forward) during the pretest (z = 2.040, *p* = 0.041). Likewise, higher ARs were seen in the TD group with intervention in the mismatch future condition (i.e., future–backward) compared to the matching past condition (i.e., past–backward) (z = 3.165, *p* = 0.002).

After the intervention, higher accuracy rates were recorded in the mismatch past condition compared to the matching future condition (z = 2.078, *p* = 0.038) in the DLD group with intervention. In the TD group with intervention, higher accuracy rates were identified in the matching future condition compared to the mismatch past condition during the posttest (z = 2.585, *p* = 0.010)

### 3.3. Descriptive Statistics

Descriptive statistics were estimated for the past and future verbs for the DLD groups with intervention (Table 3) and without intervention (Table 4). Overall, both groups showed slower RTs in the posttests for past tense verbs, although the group with intervention was slightly faster than the group without intervention when understanding future tense verbs during the posttest. Likewise, the DLD group with intervention showed an increase in ARs during the posttest for both past- and future-tense verbs.

In addition to the above, the descriptive statistics for the future- and past-tense verbs were estimated for the TD groups with (Table 5) and without intervention (Table 6). Both groups were faster and more accurate in their responses during the posttest and showed more accurate results for future-tense verbs after the intervention.

When comparing the results of all four groups, the data confirm that the TD groups (both with and without intervention) had faster results in comprehending past and future tense in the posttest compared to the DLD groups (both with and without intervention).

### 3.4. Analysis of Reaction Times with Log Transformation

Repeated measures ANOVA showed a significant four-way group*intervention*verb semantics*motion interaction, *F* (1, 1000) = 5.089, *p* = 0.028, η^2^ = 0.088, β = 0.601. Likewise, a significant four-way group*intervention*motion*session interaction, *F*(1, 1000) = 5.640, *p* = 0.021, η^2^ = 0.096, β = 0.645. In the two-way interactions, the motion*intervention interaction was significant *F*(1, 1000) = 8.67, *p* = 0.005, η^2^ = 0.141, β = 0.824, as well as the group*session interaction, *F*(1, 1000) = 13.326, *p* < 0.005, η^2^ = 0.201, β = 0.948. Finally, a significant main effect was observed for the within-subject session variable, *F*(1, 100) = 12.453, *p* < 0.005, η^2^ = 0.190, β = 0.934, where posttest RTs were faster (M = 3.067) compared to the pretest (M = 3.102).

No significant results were found for the group*intervention*verb semantics*motion*session interaction, *F*(1, 1000) = 0.009, *p* = 0.923, η^2^ = 0.001. No significant main effects were found for either the between-subject group variable, *F*(1, 1000) = 0.063, *p* = 0.803, η^2^ = 0.001, or the intervention variable, *F*(1, 1000) = 0.056, *p* = 0.814, η^2^ = 0.001. Likewise, the main effects for the within-subject variable verb semantics (*F*(1, 1000) = 0.253, *p* = 0.617, η^2^ = 0.005) and motion (*F*(1, 1000) = 1.463, *p* = 0.232, η^2^ = 0.027) were not significant.

In the *post hoc* analysis of marginal means, the TD group showed no significant results in the pretest and posttest sessions, as opposed to the DLD group, which did show statistically significant results in the posttest. Bonferroni corrections were applied to adjust for multiple comparisons.

The DLD group results showed a facilitation effect in the matching condition of past*backward motion in the posttest. This was reflected in a statistically significant negative difference between RTs of the group with intervention versus the group without intervention: *Mdiff* = −0.124, *p* = 0.034, IC 95% [−0.238, −0.010]. In the matching condition future–forward motion, an interference effect was found. This was reflected by a significant positive difference between RTs both in the group with intervention and the group without intervention: *Mdiff* = 0.159, *p* = 0.020, IC 95% [0.026, 0.291].

**Figure 5:**
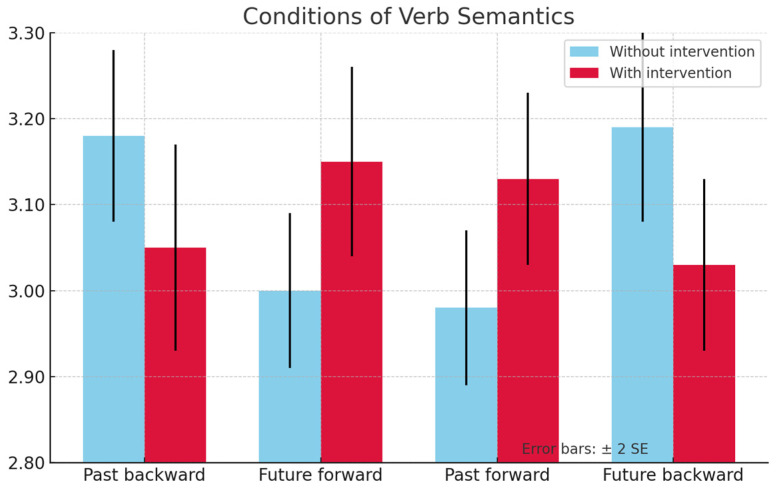
Graph of RT results in the posttest for the DLD group.

**Figure 6:**
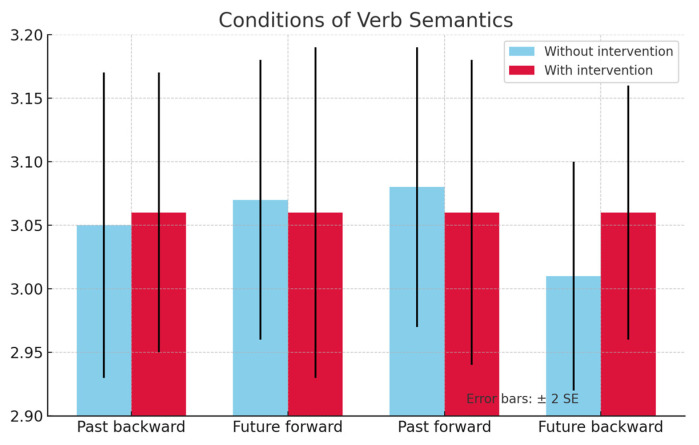
Graph chart of RT results in the posttests for the TD group.

Although the results of the TD group in the posttest were not significant, they were considerably different from those of the DLD group and were taken as a foundation for the analysis of the latter. On the one hand, Figure 5 shows a facilitation effect of ‘past–backward’ and an interference effect of ‘future–forward’ in the DLD group with intervention. On the other hand, Figure 6 shows the results of the DLD group with a trend towards interference for the ‘past–backward’ condition and towards facilitation in the ‘future–forward’ condition when comparing the group with intervention versus the group without intervention during the posttest.

#### Interaction Between Covariable and Reaction Times

The effects of the covariables ‘selective attention’, ‘inhibitory control’, ‘working memory’, and ‘narrative discourse comprehension’ were analyzed. However, no modulation effects were found for these covariables.

### 3.5. Analysis of Accuracy Rates with Log Transformation

Repeated measures ANOVA showed a significant four-way group*intervention*motion*session interaction, *F*(1, 1000) = 4.186, *p* = 0.046, η^2^ = 0.073, β = 0.520. A significant main effect of the between-subject variable group was found, *F*(1, 53) = 6.748, *p* = 0.012, η^2^ = 0.113, β = 0.723. Likewise, a significant main effect of the within-subject variable verb semantics was found, *F*(1, 1000) = 12.326, *p* < 0.005, η^2^ = 0.189, β = 0.931, in which ARs for verbs in past tense (M = 1.592) was lower than ARs for verbs in future tense (M = 1.620). A significant main effect was found for the variable session, *F*(1, 1000) = 14.916, *p* < 0.001, η^2^ = 0.220, β = 0.966, whereby ARs for the posttest were higher (M = 1.629) than for the pretest (M = 1.583).

No significant results were found for the group*intervention*verb semantics*motion*session interaction, *F*(1, 1000) = 0.038, *p* = 0.846, η^2^ = 0.001. No significant main effects were found for the variables intervention, *F*(1, 1000) = 0.836, *p* = 0.365, η^2^ = 0.016, or motion, *F*(1, 1000) = 1.461, *p* = 0.232, η^2^ = 0.027.

In the *post hoc* analysis of marginal means, the TD group showed no significant results in the pretest and posttest sessions. Conversely, the DLD group showed statistically significant differences in the posttest between the with intervention and the without intervention groups. In the future-matching condition, there was a facilitation effect, i.e., a significant increase in participants’ ARs in the group with intervention when compared to the ARs in the group without intervention, *Mdiff* = 0.141, *p* = 0.022, IC 95% [0.021, 0.260]. Bonferroni corrections were applied to adjust for multiple comparisons.

**Figure 7:**
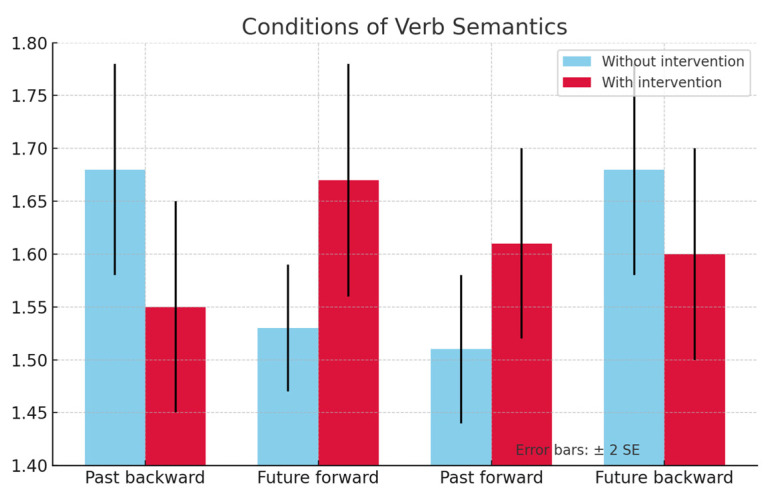
Graph of AR results in the posttests for the DLD group.

**Figure 8:**
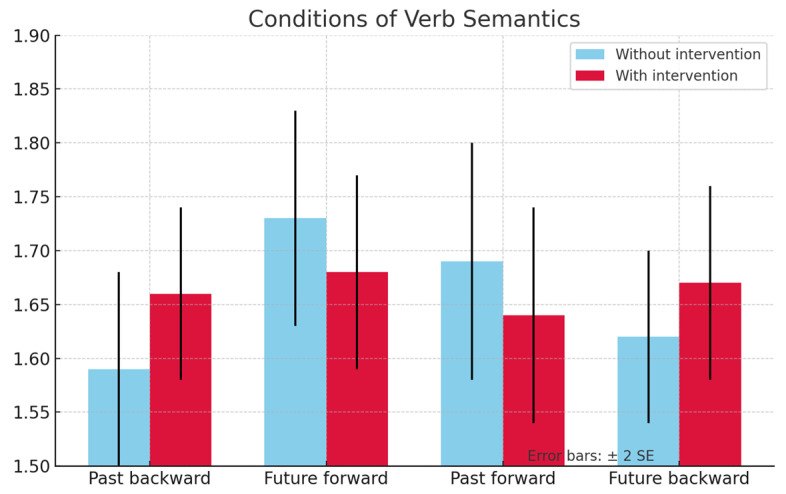
Graph of AR results in the posttests for the TD group.

Figure 7 shows DLD group results in the posttest and Figure 8 shows the results of the TD group in the posttest. In both cases, what is more noteworthy is that in the group with intervention, the interference effect occurs in the past-matching condition, when compared to the future-matching condition.

#### Interaction Between Covariate and Accuracy Rates

In the DLD group, the inclusion of the covariate ‘comprehension of narrative discourse’ led to a significant interaction with motion and session: *F*(1, 1000) = 5.458, *p* = 0.027, η^2^ = 0.174. The *t*-test showed significant differences in the matching condition for past–backward motion both in the pretest (*t*(27) = −2.231, *p* = 0.034) and the posttest (*t*(27) = 2.304, *p* = 0.029).

‘Comprehension of narrative discourse’ was the only covariate with significant effects after the intervention. In the posttest, the *t*-test of the DLD group with intervention showed a facilitation effect when comprehension was higher (M = 1.644) and an interference effect when comprehension was lower (M = 1.470), *t*(12) = 2.558, *p* < 0.025) for the matching condition for past–backward movement.

The ‘inhibitory control’ covariate also led to a significant interaction with motion and session (*F*(1, 1000) = 9.905, *p* = 0.004), as well as significant differences in the mismatch condition for past–forward motion: *t*(27) = −2.118, *p* = 0.044. In the DLD group with intervention, the T-test showed a facilitation effect when inhibitory control was normal (M = 1.565) in contrast to an interference effect when inhibitory control was low (M = 1.462). All other variables showed no significant differences.

## 4. Discussion

This study provides empirical data on the comprehension of verb tense in TD children and children with DLD by using the ‘TIME IS SPACE’ metaphor. This assumes that future is forward and past is backward from an embodied point of view. We carried out a psychoeducational intervention based on embodiment, and its effects were evaluated through a behavioral experiment involving induced plasticity (Casasanto and de Bruin 2019; Casasanto and Chrysikou 2011; Ruiz et al. 2021), both before and after the intervention.

Repeated-measures ANOVA showed that verb semantics, matching motion, session, intervention, and group had significant interactions, affecting both the participants’ RTs and ARs in the induced plasticity experiment. In particular, the study showed that the intervention had an influence on the DLD group, leading to an improvement in performance when processing verbs. This improvement was seen when the verbs were compatible with the ‘TIME IS SPACE’ metaphor in experimental conditions. In the TD group, no clear effects were found in relation to the intervention. This suggests that high levels of reading comprehension are not significantly influenced by the compatibility of actions and verbs or by the impact of the intervention. However, these results may be subject to a more specific interpretation that will be addressed in this discussion.

In this context, the primary hypothesis suggested that children with DLD would show improved comprehension of conceptual metaphors related to time and space following a psychoeducational intervention. This improvement should be reflected by an interference effect: higher RTs and lower ARs in matching conditions for verb semantics–motion, as opposed to mismatch conditions.

The results partially confirmed this hypothesis. While interference effects were clearly observed, particularly in matching conditions involving future-tense verbs, other outcomes diverged from the predicted pattern. Specifically, children with DLD who received the intervention demonstrated facilitation effects in matching conditions, particularly in reaction times (RTs) for past-tense verbs. This pattern suggests that the integration between language and action may not yet be fully consolidated in this population.

Bidet-Ildei et al. (2022) reported significant differences related to the age of embodied representations of actions mediated by language. The study revealed that TD children aged 7 to 8 significantly benefited from matching action verbs with corresponding images depicting those actions. Conversely, no significant differences were found for children aged 5 to 6 years either in the matching or mismatch conditions. According to the authors, this lack of spontaneous activation of the sensory–motor representation in younger children evidences that the link between language and action is progressively strengthened at around 7 years old.

The ANOVA results showed that RTs in the DLD group with intervention were higher in the ‘future–forward’ conditions, while RTs were lower in the ‘past–backward’ condition after the intervention. This difference between responses to matching conditions might be due to biomechanical differences between forward and backward motion. In a dual-task paradigm, Marrero et al. (2015) showed that backward motion requires more time and effort than forward motion. This suggests that the egocentric posture has an effect on cognition.

It is possible that less difficult forward motion facilitates automation of embodied metaphors in the ‘future–forward’ matching condition, which would cause the expected interference effect. In contrast, the higher difficulty of backward motion in the past–backward matching condition makes systemic integration of embodied metaphors more difficult, leading to an unexpected facilitation effect.

Casasanto et al. (2010) support the idea that the estimation of time and distance is not addressed asymmetrically in children. As a result, there is a higher accuracy of distance when there is temporal interference, as opposed to spatial interference. The asymmetry hypothesis emerges from metaphorical language patterns in which motor perceptual experiences might play an important role in the representation of actions. Thus, it is likely that interference might be latent in children with worse reading comprehension, as their sensory–motor experience is less enriched. That is not the case for children with higher reading comprehension, who show facilitation in the experiment, maybe due to being more experienced with sensory–motor patterns acquired through narration (Iossifova and Marmolejo-Ramos 2013).

The relation between sensory–motor and narrative comprehension is also reflected in the effects seen in this study. In the analysis of the ‘comprehension of narrative discourse’ covariate, we found a significant impact of narrative comprehension levels on ARs of past-tense verbs in the matching condition, specifically in the DLD group with intervention in the posttest, as opposed to the DLD group without intervention, who showed no statistically significant results. Participants with a higher narrative comprehension showed a facilitation effect (higher ARs) in the processing of past verbs matching the ‘TIME IS SPACE’ metaphor, while those with a lower narrative comprehension showed an interference effect (lower ARs).

These results might be explained by the integration of mechanisms for embodied metaphors in children with DLD, who have different levels of narrative discourse comprehension. For participants with a higher level, the integration of a metaphor does not exclusively depend on the immediate motor experience of the induced plasticity experimental task. Rather, integration benefits from the activation of previous sensory–motor experiences associated with narrative knowledge. In contrast, in children with higher narrative comprehension, the ‘TIME IS SPACE’ metaphor is internalized mainly through the immediate experience of the motor task of the experiment and to a lesser degree through previous sensory–motor experiences associated with narrations.

In this regard, Iossifova and Marmolejo-Ramos (2013) showed that sensory–motor experience plays a key role in the processing of spatial and temporal concepts. In their study, children with visuomotor impairments (strabismus and/or amblyopia) showed less automatic sensory–motor activation compared to sighted children, affecting their capacity to process abstract concepts through motor action. In blind children, the lack of visual information leads to a reorganization of the sensory–motor system, causing them to depend exclusively on touch, movement, and proprioception to carry out directional or temporal deixis tasks.

The results of Trevisan et al. (2017) also support the theory that language comprehension is closely related to individual sensory–motor experience. The authors’ study showed that training through video games with bodily interaction selectively improved comprehension of narrative discourse in children with dyslexia. Similarly, Feng and Zhou (2021) found that less competent speakers have an initial activation of sensory–motor information associated with concrete words of a metaphor before understanding their abstract meaning. However, more competent speakers tend to categorize metaphors more directly, without resorting to sensory–motor representations during their initial processing (Al-Azary and Katz 2021).

Sensory–motor experiences can strengthen the activation of narrative schemes by providing concrete references, enabling comprehension and structuring of discourse. This is why DLD participants with higher scores in narrative comprehension show a more efficient activation of these schemes, as they have more perceptual and motor experience. This allows them to adequately organize the content of discourse, improve the formulation of inferences, and comprehend the meaning of the text (Delgado-Cruz et al. 2024). In contrast, participants with lower narrative comprehension do not spontaneously activate such schemes during processing of more complex meaning, such as past-tense verb semantics, because they have less perceptual and motor experience. Consequently, children with DLD and lower narrative comprehension show interference effects in ARs in the past-matching condition, as opposed to the effect seen on children with DLD and higher narrative comprehension.

Likewise, based on the ANOVA results for the DLD group with intervention and the nonparametric test results for the TD group with intervention, the analysis of accuracy rates (ARs) revealed a significant facilitation effect in the comprehension of future-tense verbs under conditions matching the ‘TIME IS SPACE’ metaphor. This facilitation pattern aligns with the notion that the difficulties inherent to DLD inhibit the overall effectiveness of interventions (Finestack 2018; Acosta et al. 2017). A related finding was reported in the study by Dam et al. (2020), in which cognate-based interventions led to improvements in specific areas, but failed to fully overcome the linguistic barriers faced by children with DLD.

The facilitation effect in the DLD group with intervention is consistent with the results reported by Ruiz et al. (2021). According to the authors, children with DLD resort to embodied metaphors in order to facilitate their linguistic comprehension differently to the TD group. The behavioral response of facilitation in metaphorical processing of the future tense might reflect a dysregulation of the cognitive system and can be the result of an inherent characteristic of deficits in DLD.

In the same vein, neuroimaging and TMS studies (Albouy et al. 2016; Censor et al. 2014a, 2014b) have reported that the facilitation and interference effects share activity and functional connectivity in several key areas of the motor system. Relevant ones include corticostriatal connectivity: a network that is affected in DLD (Herszage and Censor 2018) and plays a key role in motor control and procedural memory. Cognitive dysregulation in children with DLD can be evidenced differently in verbs in future and in past tense as a result of the morphological and semantic differences in the verbs.

One limitation of this study was the duration of the intervention, as it could have benefited from a longer time of application. This would have provided more time to analyze significant changes in language comprehension in young children who have language difficulties in relation to past tense. Future studies should explore how various sensory–motor and perceptual strategies can be integrated into longer-term interventions. This could allow researchers to assess the long-term effects of such interventions.

Another promising area of research is the influence of other conceptual metaphors and multidimensional approaches on language comprehension in children with DLD. By using other methodologies for this type of study, such as electrophysiology and longitudinal studies, researchers could gain a deeper understanding of the mechanisms underlying the development of the relationship between language and action in this population. Future studies could explore the expansion of the mobile app by incorporating complementary, movement-based didactic strategies that promote active engagement. These strategies could help counteract sedentary behavior in young children by integrating physical activity into learning tasks, further supporting the embodied nature of language processing.

In summary, the effects observed in our study highlight the interaction between the characteristics of DLD, narrative comprehension, and embodied interventions. Children with DLD who have lower narrative comprehension seem to depend more on immediate motor or perceptual experience of spatial displacement. Conversely, children with DLD who have higher narrative comprehension activate narrative and sensory–motor schemes that enable inference formulation and the integration of complex meaning, particularly for past-tense verbs. These findings highlight the need to design personalized interventions that consider the variability of narrative skills, which are paramount when addressing specific deficits of DLD.

## 5. Conclusions

The results of this study offer an encouraging view on the potential of psychoeducational interventions based on embodiment to improve comprehension of verb tenses in children with DLD. Although we expected to see an interference pattern (i.e., higher RTs and lower ARs in the posttest), the intervention showed to be able to generate positive effects on verb processing, particularly in matching conditions by using the ‘TIME IS SPACE’ metaphor. While RTs in the ‘past–backward’ condition might be related to different factors, such as biomechanical movement, egocentric posture of participants, the difficulty of the task, or the lack of consolidation of the link between language and action, the results also reflect that children with DLD can benefit from learning strategies that include embodiment.

Comprehension of narrative discourse arose as a valuable resource in the posttest, as it provides an explanation for the performance of children who participated in the embodied intervention of induced plasticity. In this study, participants with a higher comprehension level had more accurate results. This suggests that the intervention can be especially effective when the specific linguistic profile of children is known.

Similarly, our results highlight the value of creating more personalized approaches tailored to children with language disorders. In this regard, it is crucial to explore new strategies that strengthen the link between language and embodied experience. Adapting interventions to individual characteristics can maximize their effectiveness and help children with DLD overcome challenges related to semantic processing.

## Figures and Tables

**Table 1:** Participants’ description.

Group		Age	Min-Max	SD	N	t	*p*
DLD	With intervention	6.143	5.7–6.7	0.354	14	1418	0.168
	Without intervention	5.960	5.7–6.7	0.339	15		
TD	With intervention	6136	5.9–6.9	0.397	14	−3268	<0.005
	Without intervention	6629	5.6–6.9	0.400	14		

***Note:*** The table includes the number of participants per group, their average age (including date of birth: month and year), and standard deviation for groups with DLD with intervention, DLD without intervention, TD with intervention and TD without intervention. Source: prepared by the authors.

**Table 2:** Control variables.

Variable	Group	Intervention	Mean	t	*p*
RLI	DLD	1	74,285	1004	0.324
		0	70,666		
	TD	1	80,500	−0880	0.387
		0	84,714		
LCI	DLD	1	76,428	0969	0.341
		0	72,933		
	TD	1	82,428	−0092	0.927
		0	82,785		
Non-Verbal IQ	DLD	1	55	1871	0.084
		0	50		
	TD	1	59,642	−0912	0.370
		0	64,285		

***Note:*** The table shows values of control variables: Receptive Language Index (RLI), Linguistic Context Index (LCI) and non-verbal IQ. The results are shown by group: children with DLD and TD in two conditions: with (value = 1) or without intervention (value = 0). T-test results show no significant differences between DLD and TD groups.

**Table 3:** Descriptive statistics of the DLD group with intervention.

DV	Verb	Session	Min	Max	Mean	SD
RT	Past	1	982	3481	173,164	658,291
	Future	1	967	2254	1695.16	447,487
	Past	2	1058	2529	1778.63	439,951
	Future	2	1215	2407	1686.61	346,782
AR	Past	1	17%	83%	46.93%	20,458%
	Future	1	22%	89%	54.79%	23,371%
	Past	2	17%	89%	49.21%	18,394%
	Future	2	33%	78%	55.50%	14,195%

***Note:*** The table includes minimum and maximum ranges, the mean and standard deviation (SD) for the dependent variable (DV), reaction times (RTs), and accuracy rates (ARs) for verbs in past and future tense in the pretest (1) and posttest (2) in the DLD group with intervention. Source: prepared by the authors.

**Table 4:** Descriptive statistics of the DLD group without intervention.

DV	Verb	Session	Min	Max	Mean	SD
RT	Past	1	1040	2368	1553.76	449,802
	Future	1	934	2447	1561.60	504,831
	Past	2	1095	2393	1710.75	379,170
	Future	2	1337	2742	1810.97	454,093
AR	Past	1	11%	78%	54.87%	17,928%
	Future	1	17%	83%	54.87%	21,993%
	Past	2	22%	89%	54.53%	16,361%
	Future	2	22%	100%	53.67%	19,223%

***Note:*** Source: prepared by the authors.

**Table 5:** Descriptive statistics of the DT group with intervention.

DV	Verb	Session	Min	Max	Mean	SD
RT	Past	1	1037	2945	1762.24	582,488
	Future	1	1005	2756	1804.98	554,642
	Past	2	734	2113	1502.65	451,952
	Future	2	870	2646	1475.78	456,385
DV	Past	1	11%	83%	46.86%	20,373%
	Future	1	28%	94%	56.43%	21,436%
	Past	2	33%	94%	66.64%	16,108%
	Future	2	33%	100%	68.93%	19,012%

***Note:*** The table includes minimum and maximum ranges, the mean, and standard deviation (SD) for the dependent variable (DV), and for verbs in past and future tense in the pretest (1) and posttest (2) in the TD group with intervention. Source: prepared by the authors.

**Table 6:** Descriptive statistics of the TD group without intervention.

DV	Verb	Session	Min	Max	Mean	SD
RT	Past	1	945	2495	1842.27	482,168
	Future	1	1140	3248	1942.38	610,983
	Past	2	787	2622	1519.34	529,384
	Future	2	718	3014	1485.15	622,639
DV	Past	1	33%	89%	62.64%	18,529%
	Future	1	28%	83%	61.93%	15,449%
	Past	2	28%	94%	63.93%	20,163%
	Future	2	44%	94%	69.36%	16,364%

***Note:*** Source: prepared by the authors.

## Data Availability

The data generated and analyzed in this study are available on reasonable request from the corresponding author. The data are not publicly available, as they are human data from adults and children in neurotypical and clinical groups.

## References

[B1-jintelligence-13-00061] Acosta Víctor, Ramírez Gustavo, Hernández Sergio (2017). Funciones ejecutivas y lenguaje en subtipos de niños con trastorno específico del lenguaje. Neurología.

[B2-jintelligence-13-00061] Acosta Víctor, Ramírez Gustavo, Valle Nayarit del, Castro Laura de (2016). Intervention in Reading Processes in Pupils with Specific Language Impairment (SLI). Psicothema.

[B3-jintelligence-13-00061] Ahufinger Nadia, Berglund-Barraza Amy, Cruz-Santos Anabela, Ferinu Laura, Andreu Llorenç, Sanz-Torrent Mónica, Evans Julia (2021). Consistency of a Nonword Repetition Task to Discriminate Children with and without Developmental Language Disorder in Catalan–Spanish and European Portuguese Speaking Children. Children.

[B4-jintelligence-13-00061] Al-Azary Hamad, Katz Albert (2021). Do metaphorical sharks bite? Simulation and abstraction in metaphor processing. Memory & Cognition.

[B5-jintelligence-13-00061] Albouy Geneviève, King Bradley, Schmidt Christina, Desseilles Martin, Dang-Vu Thien Thanh, Balteau Evelyne, Phillips Christophe, Degueldre Christian, Orban Pierre, Benali Habib (2016). Cerebral Activity Associated with Transient Sleep-Facilitated Reduction in Motor Memory Vulnerability to Interference. Scientific Reports.

[B6-jintelligence-13-00061] American Psychiatric Association (APA) (2013). Diagnostic and Statistical Manual of Mental Disorders.

[B7-jintelligence-13-00061] Andreou Georgia, Lymperopoulou Vasiliki, Aslanoglou Vasiliki (2022). Developmental Language Disorder (DLD) and Autism Spectrum Disorder (ASD): Similarities in Pragmatic Language Abilities. A Systematic Review. Research in Autism Spectrum Disorders.

[B8-jintelligence-13-00061] Andreu Llorenç, Ahufinger Nadia, Igualada Alfonso, Sanz-Torrent Mónica (2021). Descripción del cambio del TEL al TDL en contexto angloparlante. Revista de Investigación en Logopedia.

[B9-jintelligence-13-00061] Barsalou Lawrence (2008). Grounded cognition. Annual Review of Psychology.

[B10-jintelligence-13-00061] Beracci Alessia, Fabbri Marco (2024). Vertical mental timeline is not influenced by visuo-spatial processing. Brain Sciences.

[B11-jintelligence-13-00061] Berchicci Marika, Russo Yuri, Bianco Valentina, Quinzi Federico, Rum Lorenzo, Macaluso Andrea, Committeri Giorgia, Vannozzi Giuseppe, Russo Francesco Di (2020). Stepping Forward, Stepping Backward: A Movement-Related Cortical Potential Study Unveils Distinctive Brain Activities. Behavioral Brain Research.

[B12-jintelligence-13-00061] Berger Alexander, Kiefer Markus (2021). Comparison of different response time outlier exclusion methods: A simulation study. Frontiers in Psychology.

[B13-jintelligence-13-00061] Bidet-Ildei Christel, Beauprez Sophie Anne, Badets Arnaud (2020). A Review of Literature on the Link Between Action Observation and Action Language: Advancing a Shared Semantic Theory. New Ideas in Psychology.

[B14-jintelligence-13-00061] Bidet-Ildei Christel, Beauprez Sophie Anne, Toussaint Lucette (2022). The Link Between Action Verb Processing and Action Observation: A Developmental Study. Perceptual and Motor Skills.

[B15-jintelligence-13-00061] Bishop Dorothy, Snowling Margaret, Thompson Paul, Greenhalgh Trisha, CATALISE consortium (2017). Phase 2 of CATALISE: A multinational and multidisciplinary Delphi consensus study of problems with language development: Terminology. Journal of Child Psychology and Psychiatry.

[B16-jintelligence-13-00061] Blom Elma, Boerma Tessel (2020). Do Children with Developmental Language Disorder (DLD) Have Difficulties with Interference Control, Visuospatial Working Memory, and Selective Attention? Developmental Patterns and the Role of Severity and Persistence of DLD. Journal of Speech, Language, and Hearing Research.

[B17-jintelligence-13-00061] Bühler Daniela, Perovic Alexandra, Pouscoulous Nausicaa (2018). Comprehension of Novel Metaphor in Young Children with Developmental Language Disorder. Autism & Developmental Language Impairments.

[B18-jintelligence-13-00061] Casasanto Daniel, de Bruin Angela (2019). Metaphors we learn by: Directed motor action improves word learning. Cognition.

[B19-jintelligence-13-00061] Casasanto Daniel, Chrysikou Evangelia (2011). When Left is “Right”: Motor fluency shapes abstract concepts. Psychological Science.

[B20-jintelligence-13-00061] Casasanto Daniel, Boroditsky Lera (2008). Time in the mind: Using space to think about time. Cognition.

[B21-jintelligence-13-00061] Casasanto Daniel, Fotakopoulou Olga, Boroditsky Lera (2010). Space and Time in the Child’s Mind: Evidence for a Cross-Dimensional Asymmetry. Cognitive Science.

[B22-jintelligence-13-00061] Censor Nitzan, Dayan Eran, Cohen Leonardo (2014a). Cortico-Subcortical Neuronal Circuitry Associated with Reconsolidation of Human Procedural Memories. Cortex.

[B23-jintelligence-13-00061] Censor Nitzan, Horovitz Silvina, Cohen Leonardo (2014b). Interference with Existing Memories Alters Offline Intrinsic Functional Brain Connectivity. Neuron.

[B24-jintelligence-13-00061] Conti-Ramsden Gina, Durkin Kevin, Skuse David, Bruce Helen, Dowdney Linda (2017). Developmental Language Disorder. Child Psychology and Psychiatry: Frameworks for Clinical Training and Practice.

[B25-jintelligence-13-00061] Curran-Everett Douglas (2018). Explorations in statistics: The log transformation. Advances in Physiology Education.

[B26-jintelligence-13-00061] Dam Quynh, Pham Giang, Pruitt-Lord Sonja, Limon-Hernandez Judit, Goodwiler Carrie (2020). Capitalizing on cross-language similarities in intervention with bilingual children. Journal of Communication Disorders.

[B27-jintelligence-13-00061] Dauvister Estelle, Jemel Boutheina, Maillart Christelle (2022). Preserved Category-Based Inferences for Word Learning in School-Aged Children with Developmental Language Disorder. Clinical Linguistics & Phonetics.

[B28-jintelligence-13-00061] Dawes Emily, Leitão Suze, Claessen Mary, Kane Robert (2019). A randomized controlled trial of an oral inferential comprehension intervention for young children with developmental language disorder. Child Language Teaching and Therapy.

[B32-jintelligence-13-00061] de Koning Björn, Bos Lisanne, Wassenburg Stephanie, Schoot Menno van der (2017). Effects of a reading strategy training aimed at improving mental simulation in primary school children. Educational Psychology Review.

[B33-jintelligence-13-00061] de Vega Manuel (2021). Revisitando la Corporeidad del Lenguaje Narrativo. Revista Signos.

[B29-jintelligence-13-00061] Delgado-Cruz Atteneri, Acosta-Rodríguez Víctor, Ramírez-Santana Gustavo (2022). Intervention in the Coherence of Narrative Discourse in Students With Developmental Language Disorder and With Typical Development. Journal for the Study of Education and Development.

[B30-jintelligence-13-00061] Delgado-Cruz Atteneri, Acosta-Rodríguez Víctor, Ramírez-Santana Gustavo (2024). Enseñanza de Esquemas Narrativos en Alumnado de Educación Infantil: Trastorno del Desarrollo del Lenguaje y Desarrollo Típico. Estudios Sobre Educación.

[B31-jintelligence-13-00061] Demir Özlem Ece, Goldin-Meadow Susan, Hickok Gregory, Small Steven (2016). Gesture’s role in learning and processing language. Neurobiology of Language.

[B34-jintelligence-13-00061] Faul Franz, Erdfelder Edgar, Buchner Axel, Lang Albert Georg (2009). Statistical Power Analyses Using G*Power 3.1: Tests for Correlation and Regression Analyses. Behavior Research Methods.

[B35-jintelligence-13-00061] Feng Yin, Zhou Rong (2021). Does embodiment of verbs influence predicate metaphor processing in a second language? Evidence from picture priming. Frontiers in Psychology.

[B36-jintelligence-13-00061] Finestack Lizbeth (2018). Evaluation of an Explicit Intervention to Teach Novel Grammatical Forms to Children with Developmental Language Disorder. Journal of Speech, Language, and Hearing Research.

[B37-jintelligence-13-00061] Gallese Vittorio (2014). Bodily Selves in Relation: Embodied Simulation as Second-Person Perspective on Intersubjectivity. Philosophical Transactions of the Royal Society B: Biological Sciences.

[B38-jintelligence-13-00061] García-García José Antonio, Reding-Bernal Arturo, López-Alvarenga Juan Carlos (2013). Cálculo del tamaño de la muestra en investigación en educación médica. Investigación en Educación Médica.

[B39-jintelligence-13-00061] Gijssels Tom, Casasanto Daniel, Dancygier Barbara (2017). Conceptualizing Time in Terms of Space: Experimental Evidence. Cambridge Handbook of Cognitive Linguistics.

[B40-jintelligence-13-00061] Glenberg Arthur, Kaschak Michael (2002). Grounding language in action. Psychonomic Bulletin and Review.

[B41-jintelligence-13-00061] Glenberg Arthur, Sato Marc, Cattaneo Luigi (2008). Use-Induced Motor Plasticity Affects the Processing of Abstract and Concrete Language. Current Biology.

[B42-jintelligence-13-00061] Hadley Pamela (2020). Exploring sentence diversity at the boundary of typical and impaired language abilities. Journal of Speech, Language, and Hearing Research.

[B43-jintelligence-13-00061] Harris Albert (1957). Lateral Dominance, Directional Confusion, and Reading Disability. The Journal of Psychology.

[B44-jintelligence-13-00061] Herszage Jasmine, Censor Nitzan (2018). Modulation of Learning and Memory: A Shared Framework for Interference and Generalization. Neuroscience.

[B45-jintelligence-13-00061] Iossifova Rositsa, Marmolejo-Ramos Fernando (2013). When the Body Is Time: Spatial and Temporal Deixis in Children with Visual Impairments and Sighted Children. Research in Developmental Disabilities.

[B46-jintelligence-13-00061] Johnson-Glenberg Mina, Megowan-Romanowicz Colleen (2017). Embodied Science and Mixed Reality: How Gesture and Motion Capture Affect Physics Education. Cognitive Research: Principles and Implications.

[B47-jintelligence-13-00061] Johnson-Glenberg Mina, Megowan-Romanowicz Colleen, Birchfield David, Savio-Ramos Caroline (2016). Effects of Embodied Learning and Digital Platform on the Retention of Physics Content: Centripetal Force. Frontiers in Psychology.

[B48-jintelligence-13-00061] Kapa Leah, Erikson Jessie (2020). The Relationship between Word Learning and Executive Function in Preschoolers with and without Developmental Language Disorder. Journal of Speech, Language, and Hearing Research.

[B49-jintelligence-13-00061] Kemmerer David, Castillo Javier, Talavage Thomas, Patterson Stephanie, Wiley Cynthia (2008). Neuroanatomical Distribution of Five Semantic Components of Verbs: Evidence from fMRI. Brain and Language.

[B50-jintelligence-13-00061] Kosmas Panagiotis, Zaphiris Panayiotis (2020). Words in Action: Investigating Students’ Language Acquisition and Emotional Performance Through Embodied Learning. Innovation in Language Learning and Teaching.

[B51-jintelligence-13-00061] Lakoff George, Johnson Mark (1980). Conceptual metaphor in everyday language. The Journal of Philosophy.

[B52-jintelligence-13-00061] Lakoff George, Johnson Mark (1999). Philosophy in the Flesh.

[B53-jintelligence-13-00061] Larson Caroline, Kaplan David, Kaushanskaya Margarita, Weismer Susan Ellis (2020). Language and Inhibition-Predictive Relationships in Children with Language Impairment Relative to Typically Developing Peers. Journal of Speech, Language, and Hearing Research.

[B54-jintelligence-13-00061] Leonard Laurence (2014). Specific language impairment across languages. Child Development Perspectives.

[B55-jintelligence-13-00061] Leonard Laurence, Karpicke Jeffrey, Deevy Patricia, Weber Christine, Christ Sharon, Haebig Eileen, Souto Sofía, Kueser Justin, Krok Windi (2019). Retrieval-Based Word Learning in Young Typically Developing Children and Children With Developmental Language Disorder I: The Benefits of Repeated Retrieval. Journal of Speech, Language, and Hearing Research.

[B56-jintelligence-13-00061] Lin Yuanyuan, Sheng Li, Shi Huanhuan, Yan Wenjie, Zhang Yiwen (2024). Narrative Generation and Narrative Recall Recruit Different Executive Functions in Preschoolers with and without Developmental Language Disorder. Clinical Linguistics & Phonetics.

[B57-jintelligence-13-00061] Lockman Jeffrey, Tamis-LeMonda Catherine (2020). Manual de Cambridge Sobre el Desarrollo Infantil: Cerebro, Comportamiento y Contexto Cultural.

[B58-jintelligence-13-00061] Loeffler Jonna, Raab Markus, Cañal-Bruland Rouwen (2020). Let’s Do the Time Warp Again: Embodied Learning of the Concept of Time in an Applied School Setting. Interactive Learning Environments.

[B60-jintelligence-13-00061] Marrero Hipólito, Gámez Elena, Díaz José, Urrutia Mabel, Vega Manuel de (2015). Carefully Encoding Approach/Avoidance Body Locomotion with Interpersonal Conduct in Narrated Interactions. Canadian Journal of Experimental Psychology.

[B61-jintelligence-13-00061] Marrero Hipólito, Yagual Sara, Díaz José, Gámez Elena, Lemus Alejandro, Urrutia Mabel, Nuez Aarón, Beltrán David (2023). Embodied Representation of Approach and Avoidance Attitudes by Language: Pro is Forward, Against is Backward. Adaptive Behavior.

[B59-jintelligence-13-00061] McGregor Karla, Pomper Ron, Eden Nichole, Appenzeller Margo, Arbisi-Kelm Timothy, Polese Elaina, Reed Deborah (2024). Inferring Word Class and Meaning from Spoken and Written Texts: A Comparison of Children with and Without Developmental Language Disorder. Journal of Speech, Language, and Hearing Research.

[B62-jintelligence-13-00061] Núñez Rafael, Cooperrider Kensy (2013). The Tangle of Space and Time in Human Cognition. Trends in Cognitive Sciences.

[B63-jintelligence-13-00061] Pavez María Mercedes (2008). Test para la Comprensión Auditiva del Lenguaje de E. Carrow.

[B64-jintelligence-13-00061] Pavez María Mercedes (2010). Test Exploratorio de Gramática Española de A. Toronto.

[B65-jintelligence-13-00061] Pavez María Mercedes, Coloma Carmen Julia, Maggiolo Mariangela (2008). El Desarrollo Narrativo en Niños: Una Propuesta Práctica para la Evaluación y la Intervención en Niños con Trastorno del Lenguaje.

[B66-jintelligence-13-00061] Pavez María Mercedes, Maggiolo Mariangela, Coloma Carmen Julia (2009). Test para Evaluar Procesos de Simplificación Fonológica.

[B67-jintelligence-13-00061] Pulvermüller Friedemann (2005). Brain Mechanisms Linking Language and Action. Nature Reviews Neuroscience.

[B68-jintelligence-13-00061] Ramírez Gustavo, Acosta Víctor, Hernández Sergio, Delgado-Cruz Atteneri (2023). Intervención en Lectura Temprana en Alumnado con Trastorno del Desarrollo del Lenguaje. Revista Signos.

[B69-jintelligence-13-00061] Raven John, McCallum R. Steve (2003). Raven Progressive Matrices. Handbook of Nonverbal Assessment.

[B70-jintelligence-13-00061] Reggin Lorraine, Gómez-Franco Ligia, Horchak Oleksandr, Labrecque David, Lana Nadia, Rio Laura, Vigliocco Gabriella (2023). Consensus Paper: Situated and Embodied Language Acquisition. Journal of Cognition.

[B71-jintelligence-13-00061] Rinaldi Sara, Caselli María Cristina, Cofelice Valentina, D’Amico Simonetta, Cagno Anna Giulia De, Corte Giuseppina Della, Martino María Valeria Di, Costanzo Brígida Di, Levorato María Chiara, Penge Roberta (2021). Efficacy of the Treatment of Developmental Language Disorder: A Systematic Review. Brain Sciences.

[B72-jintelligence-13-00061] Ruiz Daniela, Urrutia Mabel, Alarcón Paola, Marrero Hipólito (2021). Comprensión del Tiempo a Través del Espacio: Un Estudio de Plasticidad Inducida en Niños con Trastorno del Desarrollo del Lenguaje. Revista de Logopedia, Foniatría y Audiología.

[B73-jintelligence-13-00061] Sack Leah, Dollaghan Christine, Goffman Lisa (2021). Contributions of Early Motor Deficits in Predicting Language Outcomes among Preschoolers with Developmental Language Disorder. Journal of Communication Disorders.

[B74-jintelligence-13-00061] Sanjeevan Teenu, Mainela-Arnold Elina (2019). Characterizing the Motor Skills in Children with Specific Language Impairment. Folia Phoniatrica et Logopaedica.

[B75-jintelligence-13-00061] Spatola Nicolas, Santiago Julio, Beffara Brice, Mermillod Martial, Ferrand Ludovic, Ouellet Marc (2018). When the Sad Past Is Left: The Mental Metaphors Between Time, Valence, and Space. Frontiers in Psychology.

[B76-jintelligence-13-00061] Syaza-Jeffri Nor Farzana, Awang-Rambli Dayang Rohaya (2021). A Review of Augmented Reality Systems and Their Effects on Mental Workload and Task Performance. Heliyon.

[B77-jintelligence-13-00061] Tenorio-Delgado Marcela, Arango-Uribe Paulina, Aparicio-Alonso Andrés, Rosas-Díaz Ricardo (2014). TENI: A comprehensive battery for cognitive assessment based on games and technology. Child Neuropsychology.

[B78-jintelligence-13-00061] Trevisan Piergiorgio, Sedeño Lucas, Birba Agustina, Ibáñez Agustín, García Adolfo (2017). A Moving Story: Whole-Body Motor Training Selectively Improves the Appraisal of Action Meanings in Naturalistic Narratives. Scientific Reports.

[B79-jintelligence-13-00061] Wang Huili, Yan Xiaoli, Cao Shuo, Li Linxi, Kritikos Ada (2019). Interfering ACE on Comprehending Embodied Meaning in Action-Related Chinese Counterfactual Sentences. Language and Cognition.

[B80-jintelligence-13-00061] West Robert (2022). Best Practice in Statistics: The Use of Log Transformation. Annals of Clinical Biochemistry.

[B81-jintelligence-13-00061] Whelan Robert (2008). Effective Analysis of Reaction Time Data. Psychological Record.

[B82-jintelligence-13-00061] Wiig Elisabeth, Semel Eleanor, Secord Wayne (2013). Clinical Evaluation of Language Fundamentals.

[B83-jintelligence-13-00061] Winter Alice, Dudschig Carolin, Miller Jeff, Ulrich Rolf, Kaup Barbara (2022). The Action-Sentence Compatibility Effect (ACE): Meta-Analysis of a Benchmark Finding for Embodiment. Acta Psychologica.

[B84-jintelligence-13-00061] Winters Katherine, Jasso Javier, Pustejovsky James, Byrd Courtney (2022). Investigating Narrative Performance in Children With Developmental Language Disorder: A Systematic Review and Meta-Analysis. Journal of Speech, Language, and Hearing Research.

[B85-jintelligence-13-00061] Yang Ying, Dickey Michael Walsh, Fiez Julie, Murphy Brian, Mitchell Tom, Collinger Jennifer, Tyler-Kabara Elizabeth, Boninger Michael, Wang Wei (2017). Sensorimotor Experience and Verb-Category Mapping in Human Sensory, Motor and Parietal Neurons. Cortex.

[B86-jintelligence-13-00061] Zhu Rebecca, Goddu Mariel, Zhu Lily Zihui, Gopnik Alison (2024). Preschoolers’ comprehension of functional metaphors. Open Mind: Discoveries in Cognitive Science.

[B87-jintelligence-13-00061] Zwaan Rolf, Taylor Lawrence (2006). Seeing, Acting, Understanding: Motor Resonance in Language Comprehension. Journal of Experimental Psychology: General.

